# Supplementary material for: Development of an Antigen-Antibody Co-Display System for Detecting Interaction of G-Protein-Coupled Receptors and Single-Chain Variable Fragments
Source: Int J Mol Sci. 2021 Apr 29;22(9):4711. doi: 10.3390/ijms22094711 (PMC8125734; doi:10.3390/ijms22094711)
Supplement: Supplementary file 1 [file ijms-22-04711-s001.zip › ijms-1175900-supplementary.pdf]

**Supplementary Materials:**

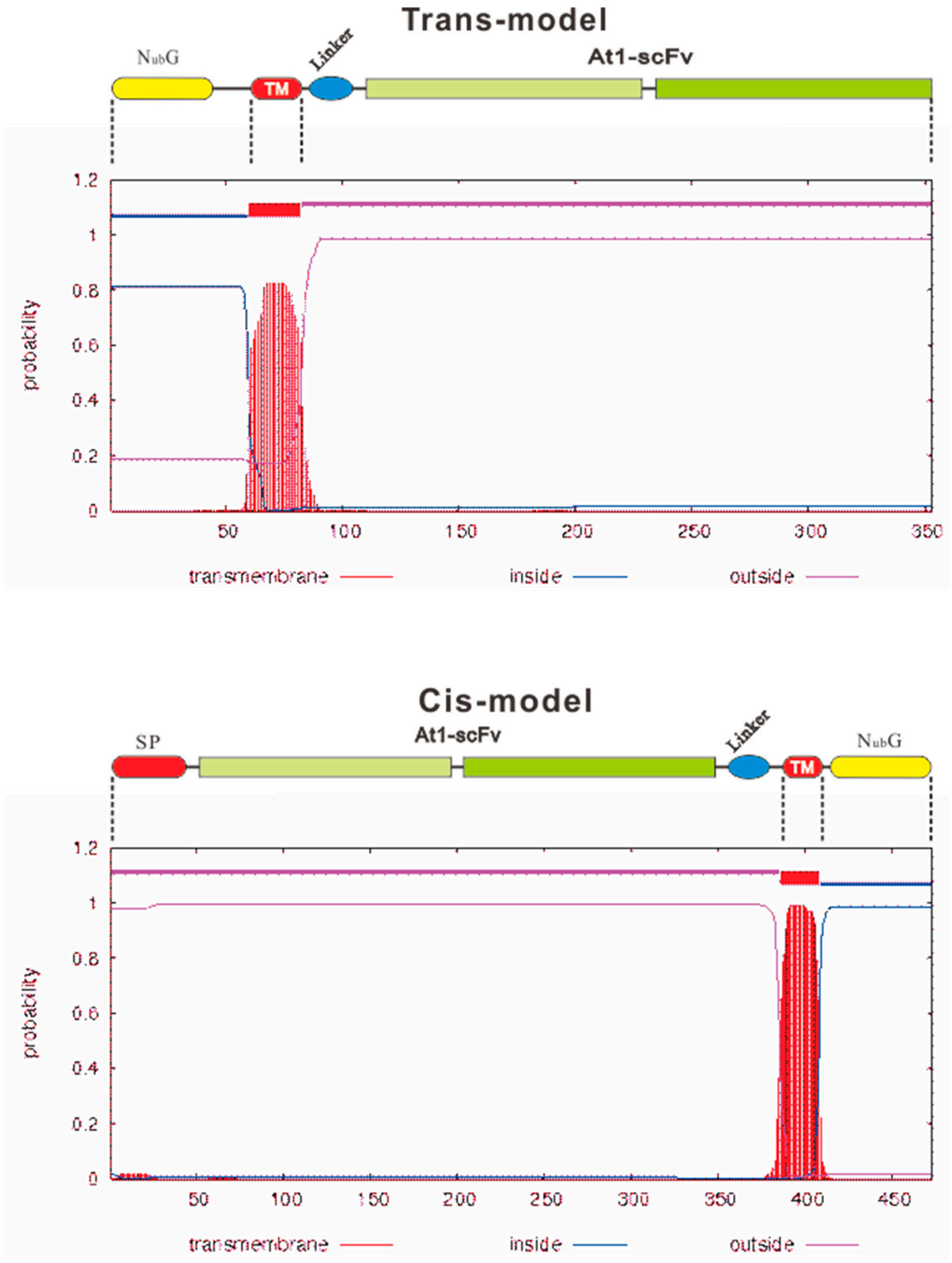


**Figure S1.** Prediction of antibody module topological structures. The topological structures of the At1-scFv antibody module with trans- or cis-model were predicted by software using the hidden Markov algorithm, TMHMM. The prediction results were consistent with our design.
